# A Bilateral Symmetrical Variant Formation of the Sural Nerve

**DOI:** 10.7759/cureus.56801

**Published:** 2024-03-24

**Authors:** George Tsakotos, George Triantafyllou, Christos Koutserimpas, Mugurel C Rusu, Răzvan Tudose, Maria Piagkou

**Affiliations:** 1 Department of Anatomy, School of Medicine, National and Kapodistrian University of Athens, Athens, GRC; 2 Department of Orthopaedics and Traumatology, 251 Hellenic Air Force General Hospital of Athens, Athens, GRC; 3 Anatomy, Faculty of Dentistry, Carol Davila University of Medicine and Pharmacy, Bucharest, ROU

**Keywords:** dissection, entrapment neuropathy, sciatic nerve, variation, sural nerve

## Abstract

Typically, the sural nerve is formatted by the connection of the lateral sural cutaneous nerve (branch of the common fibular nerve) and the medial sural cutaneous nerve (branch of the tibial nerve). The current cadaveric report aims to describe a quite unusual symmetrical variant of the sural nerve. Classical dissection was performed on an 84-year-old donated male cadaver. On both sides, the sural nerve was formatted directly by the sciatic nerve. After its emanation, it continued its typical course between the gastrocnemius muscle heads. Sural nerve formation has been extensively studied due to its great clinical significance. The identified variant corresponds to one of the rarest types of sural nerve formation. Knowledge of sural nerve variants may play a crucial role in lower limb surgery and nerve harvest for reconstruction.

## Introduction

The sciatic nerve (SCN) and the femoral nerve (FN) provide the majority of the lower limb innervation. The FN innervates the anterior thigh compartment and the SCN innervates the posterior thigh compartment and the foot and terminates as the common fibular nerve (CFN) and the tibial nerve (TN). The fibers of the SCN dorsal divisions form the CFN that innervates the lateral and anterior compartments of the foot, and the fibers of the SCN ventral division create the TN that innervates the posterior foot compartment. The two nerves give off two small sensory (cutaneous) nerves, the lateral sural cutaneous nerve (LSCN, branch of the CFN), and the medial sural cutaneous nerve (MSCN, branch of the TN), and their interconnection forms the sural nerve (SN), which courses between the gastrocnemius muscle (GM) heads, and then lateral to the calcaneal tendon along with the small saphenous vein [1]. The SN depicts great morphological variability, and its formation and origin have been meticulously studied [2,3]. Reports of nerve variations, even rare, expand the current knowledge and potentially help clinicians [4]. The current cadaveric report presents a rare variant symmetrical formation of the SN. The SN morphological variability and clinical significance are further discussed.

## Case presentation

The dissection of an 84-year-old donated male cadaver was performed. The body was donated to the Anatomy Department of the School of Medicine of the National and Kapodistrian University of Athens through the Body Donation Program after written informed consent. The cadaver was dissected, and skin, subcutaneous fat, and superficial fascia of the lower limb were removed. The small saphenous vein and the SN were identified in the superficial subcutaneous tissue and followed superiorly to the popliteal fossa, until the SCN exposure. The SN was dissected from its fascia. 

**Figure 1:**
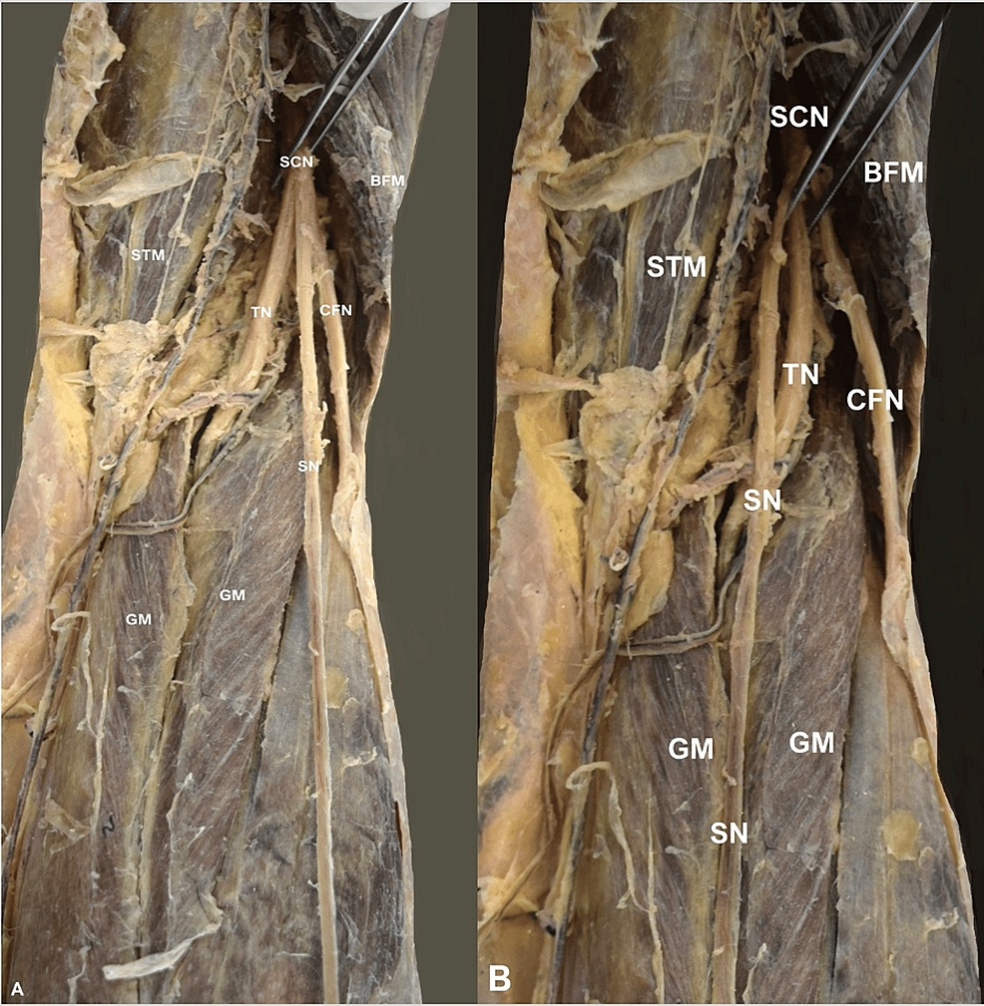
The right-sided sural nerve variant. The sciatic nerve (SCN) division into the tibial nerve (TN), the common fibular nerve (CFN), and the sural nerve (SN). GM- gastrocnemius muscle, BFM- biceps femoris muscle, STM- semitendinosus muscle.

On the right lower limb, the SN origin was visible at the popliteal fossa. It emanated directly from the SCN, along with the CFN and the TN (Figure 1A). After its formation, the SN coursed between the GM heads (Figure 1B). 

**Figure 2:**
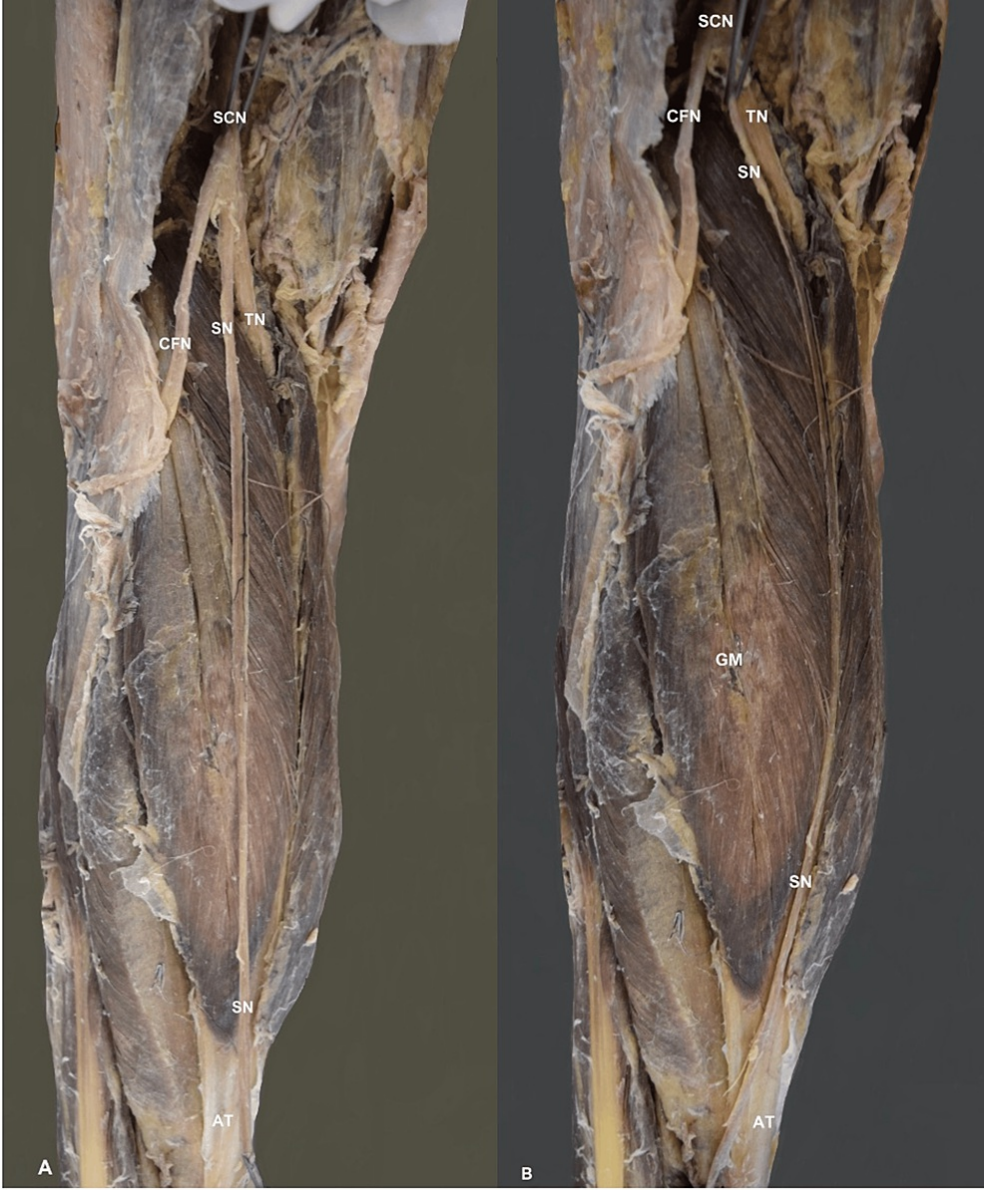
The left-sided sural nerve variant. The sciatic nerve (SCN) trifurcation into the tibial nerve (TN), the common fibular nerve (CFN), and the sural nerve (SN). GM- gastrocnemius muscle, BFM- biceps femoris muscle, STM- semitendinosus muscle, AT-Achilles tendon

On the left lower limb, the contralateral SN variant was identified, originating from the SCN at the popliteal fossa (Figure 2A). The SN continued its typical course between the GM heads (Figure 2B). The other nerves of the foot were typical. 

## Discussion

Sural nerve developmental anatomy 

Developmentally, the neural plate emerges in the third week post-fertilization and gives rise to the neural tube and crest cells. These cells develop into peripheral nerves and among them the SN. Schwann cells, which also originate from neural crest cells, are responsible for myelinating nerves within the peripheral nervous system, thereby enhancing the conduction of electrical signals [5].

Sural nerve morphological variability

In the current case, a bilateral (symmetrical) unusual origin of the SN was identified, directly from the SCN. The SN morphology and course have been extensively studied due to their great clinical significance in lower limb surgery and harvesting for reconstruction [6]. Huelke [7] was one of the first that study and classify the SN formation. He performed a cadaveric study on 352 lower limbs and identified three types of SN formation. Type A formation corresponded to the union of the MSCN (from the TN) and a peroneal communicating branch (from the CFN), identified in 80.7%. Type B SN was the continuation of the MSCN, while the peroneal communicating branch was absent (in 19%). Type C SN was formatted only from the peroneal communicating branch with or without the MSCN absence, which was identified in 0.3%. Ramakrishnan et al. [2], in their systematic review with meta-analysis, studied the pooled prevalence of SN formation variability and systematically classified its morphology. Steele et al. [3] expanded this classification, adding some unaccounted variants. According to this classification, SN of type 1 corresponds to the formation of the SN from the peroneal communicating nerve (branch of LSCN) and MSCN (51.5% pooled prevalence). In type 2, the SN is formed by the union of MSCN and LSCN (13.8%). In type 3, the SN originated from the TN (31.2%), as a continuation of the MSCN, with subtypes, the case of the LSCN absence (type 3A) and the LSCN presence with no contribution to the SN formation (type 3B). In type 4, the SN originated exclusively from the peroneal communicating nerve (1.8%). Type 5 corresponded to the SN origin from the LSCN (1.1%). In type 6, the SN was directly formed from the SCN (0.7%), as in the current case. In the present report, the bilateral (symmetrical) formation of the SN corresponds to Ramakrishnan et al. [2] and Steele et al. [3] type 6. According to Ramakrishnan et al.'s meta-analysis [2], SN symmetrical formation patterns were identified in 64.1% and asymmetrical in 35.9%. Vuksanovic-Bozaric et al. [8] reported a cadaveric case where the SN was bilaterally absent, and the MSCN and the LSCN served as cutaneous nerves of the posterior leg compartment. Steele et al. [3] classified the “absent SN” as type 7 and type 8. According to these types, the SN represents the continuation of LSCN (type 7) or MSCN (type 8). Following Ramakrishnan et al.'s classification [2], Popieluszko et al. [9] investigated the SN anatomy in an ultrasound study and identified all the types that were previously observed in cadaveric types. Therefore, ultrasound could potentially help in investigating the SN anatomy. 

Clinical significance

The SN has significance in clinical practice, encompassing both diagnostic and therapeutic realms [6]. Its extensive length and surface pathway make it a common choice for nerve grafting. According to the graft length requirements, it might be necessary to dissect up to the SN origin. Harvesting begins at the lateral malleolus level. Once the nerve is identified, it is separated and moved as proximally as needed to attain the desired length (either with open or minimally invasive techniques). Since the sensory loss is unlikely to notably escalate with a more extensive excision of the nerve, numerous surgeons routinely harvest up to 30-40 cm in length. Generally, the popliteal fossa corresponds to the endpoint of SN harvesting [6]. Knowledge of the popliteal fossa anatomy (bony landmarks and neurovascular structures) is of paramount importance for surgeons [10]. In the current report, the SN originated from the SCN, at the popliteal fossa. This rare variant could potentially complicate graft harvesting, due to its difficult origin localization. Even if this type of SN formation is considered rare (0.7%), surgeons should keep it in mind, while performing graft harvesting or procedures at the popliteal fossa to avoid iatrogenic injury. Traumatic disruption of peripheral nerves presents a common challenge encountered by plastic, orthopedic, and head and neck surgeons. While numerous injuries can and should be primarily repaired, longer defects necessitate autografts like the SN. Familiarity with indications, pertinent anatomy, and possible variants safely harvests the nerve, reducing trauma to the graft and optimizing functional outcomes [11]. In diagnostics, the SN serves essential roles in biopsy and electrophysiological studies, aiding in distinguishing peripheral neuropathies. SN iatrogenic injury has been implicated in many lower limb procedures, including internal fixation of calcaneal fractures, calcaneal osteotomies, and minimally invasive techniques of Achilles tendon repair [6,12]. Ghani et al. pointed out that the SN is at risk in the posterolateral ankle approach when treating ankle fractures. The authors studied the SN course in the posterolateral incision and concluded that in 78.5%, the nerve was at risk for injury [12]. Thus, meticulous dissection is of utmost importance for open techniques, while a careful approach to the proximal lateral area is essential for avoiding endoscopic injury. Although the SN has mainly sensory fibers, there have been reports of motor ones as well [13]. Amoiridis et al. [13] in their electrophysiological study identified SN muscular branches to abductor digiti minimi, adductor hallucis, and flexor digitorum brevis in 6.2%. SN variant origin, such as the one described in the current cadaveric report, could lead to mixed innervation. It is also of note that these variants may bilaterally occur in a symmetric way, a fact that should be kept in mind. Except for iatrogenic injury, the SN can be physically entrapped. Matsubara et al. [14] reported a case of a 67-year-old patient who presented with pain and hyperesthesia on the right foot lateral side. After neurophysiological and radiographic examination, the SN entrapment was identified. The primary cause of SN entrapment typically stems from the fascia thickening, at the point where the nerve emerges superficially to the GM, known as superficial sural aponeurosis [14,15]. Typical clinical manifestations of the SN entrapment are pain and paresthesia along the lateral ankle and foot [16]. Paraskevas et al., after dissection, identified a case where the flattening of the SN was justified after its course into a fascial tunnel [15]. Nevertheless, it has been proposed that the SN degenerates with age [17].

## Conclusions

To conclude, in the current case, a bilateral rare symmetrical formation of the SN is described. According to the current classification system, the case represents the rarest type of SN origin. Knowledge of morphological variants is important due to the SN's clinical significance.
